# Crystal structure of coagulation factor XII N-terminal domains 1–5

**DOI:** 10.1107/S2059798325005297

**Published:** 2025-06-27

**Authors:** Muhammad Saleem, Chan Li, Bubacarr G. Kaira, Alexander K. Brown, Monika Pathak, Shabir Najmudin, Nathan Cowieson, Ingrid Dreveny, Clare Wilson, Aleksandr Shamanaev, David Gailani, Stephanie A. Smith, James H. Morrissey, Helen Philippou, Jonas Emsley

**Affiliations:** a Biodiscovery Institute, School of Pharmacy University of Nottingham Nottingham United Kingdom; b Diamond Light Source Harwell Science and Innovation Campus DidcotOX11 0DE United Kingdom; c Discovery and Translational Science Department, Leeds Institute of Cardiovascular and Metabolic Medicine (LICAMM) University of Leeds Leeds United Kingdom; d Department of Pathology, Microbiology and Immunology Vanderbilt University Medical Center Nashville Tennessee USA; e Department of Biological Chemistry University of Michigan Medical School Ann Arbor Michigan USA; University of Sâo Paulo, Brazil

**Keywords:** factor XII, fibronectin type II domain, kringle domain, crystal structure

## Abstract

Factor XIIa (FXIIa) is generated from its zymogen factor XII (FXII) by contact with polyanions such as inorganic polyphosphates. FXIIa cleaves the substrates prekallikrein and factor XI, triggering inflammatory cascades and plasma coagulation. From the N-terminus, FXII has fibronectin type II (FnII), epidermal growth factor-1 (EGF1), fibronectin type I (FnI), EGF2 and kringle domains. The N-terminal domains of FXII mediate polyanion and Zn^2+^ binding. To understand how ligand binding to polyanions and Zn^2+^ is coordinated across multiple domains, we determined the crystal structure of recombinant FXII domains 1–5 (FXII^HC5^) to 3.4 Å resolution. A separate crystal structure of the isolated FXII FnII domain at 1.2 Å resolution revealed two bound Zn^2+^ ions. In FXII^HC5^ a head-to-tail interaction is formed between the FnII and kringle domains, co-localizing the lysine-binding sites of the kringle domain and the cation-binding site of the FnII domain. Two FXII^HC5^ monomers interlock, burying a large surface area of 2067 Å^2^, such that two kringle domains point outwards separated by a distance of 20 Å. The polyanion-binding site in the EGF1 domain is localized onto a plane together with the FnII and FnI domains. Using native mass spectrometry, we detected a major FXII^HC5^ monomer peak and a minor dimer peak. Small-angle X-ray scattering and gel-filtration chromatography revealed the presence of monomers and dimers in solution. These FXII N-terminal domain structures provide a holistic framework to understand how the mosaic domain structure of FXII assembles diverse ligand-binding sites in three dimensions.

## Abstract 2

Factor XII (FXII) is a plasma serine protease which becomes activated by interactions with polyanions such as polyphosphates from bacteria, with Zn^2+^ as a critical cofactor. The crystal structure of FXII domains 1–5, coupled with a second crystal structure of the isolated FnII domain in complex with Zn^2+^ ions, advances our understanding of FXII structure and ligand recognition.

## 1. Introduction

**Figure 1:**
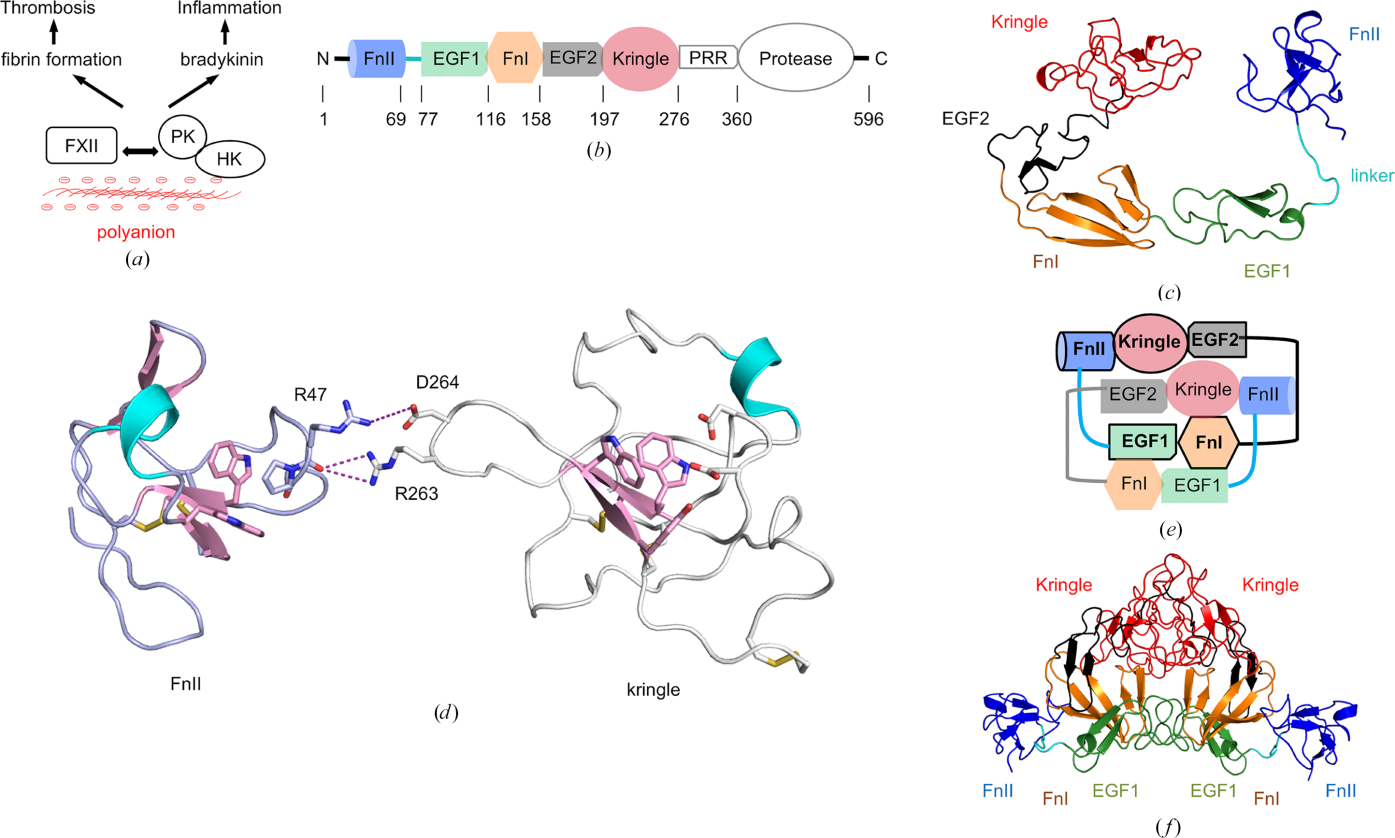
FXII function and FXII^HC5^ structure. (*a*) Contact activation is illustrated, whereby the recognition of endogenous negatively charged polymers by FXII results in reciprocal activation with prekallikrein (PK) in complex with high-molecular-weight kininogen (HK). The active enzymes trigger proteolytic cascades, resulting in inflammatory and thrombotic responses. (*b*) Schematic diagram showing the domain organization of the FXII polypeptide with the FnII domain in blue, EGF1 in green, FnI in orange, EGF2 in black, kringle in red and PRR and protease domain in white. Residue numbers are shown under each domain. (*c*) Cartoon diagram of the crystal structure of the FXII^HC5^ monomer revealing a torc shape. (*d*) The FnII and kringle domains form a head-to-tail intramolecular interaction, with key residues shown as sticks and electrostatic interactions shown as dashed lines. Residues from the cation-binding site (FnII) and the lysine-binding site (kringle) are shown as sticks. (*e*) Schematic diagram showing the dimer domain organization of the FXII^HC5^ structure. (*f*) Cartoon diagram showing the FXII^HC5^ dimer structure as two interlocking torc shapes, resulting in a triangular shape with close relative positioning of the kringle domains and distant spatial separation of the two FnII domains.

Human plasma coagulation represents a merger of two ancient systems: a thrombin-generation mechanism based on vitamin K-dependent proteases, and the contact system, which is initiated by contact of factor XII (FXII) binding to negatively charged surfaces and polymers (Ponczek *et al.*, 2008 ▸; Ivanov *et al.*, 2017 ▸; Björkqvist *et al.*, 2014 ▸). The FXII zymogen binds to polyanions such as long-chain polyphosphates (polyP) from bacteria (up to 1000 phosphate units long), resulting in auto-activation to give FXIIa. FXII autoactivation is also supported by short-chain polyP secreted from platelets (about 60–100 or 200 phosphate units long) and highly sulfated heparins secreted from mast cells, and is greatly enhanced by the presence of Zn^2+^ (Verhoef *et al.*, 2017 ▸; Wang *et al.*, 2019 ▸). FXIIa cleaves prekallikrein (PK), resulting in plasma kalli­krein (PKa) formation (Ivanov *et al.*, 2017 ▸). PKa cleaves bound high-molecular-weight kininogen (HK) resulting in the generation of the vasoactive peptide bradykinin that stimulates inflammatory cascades (Long *et al.*, 2016 ▸; Fig. 1 ▸*a*).

FXIIa generation, also termed contact activation, drives fibrin formation by cleavage of factor XI (FXI; Ivanov *et al.*, 2017 ▸). Contact activation serves as the basis of the activated partial thromboplastin time (aPTT), a method widely used to determine overall plasma coagulation in clinical practice. In plasma, FXII circulates as a single-chain polypeptide with molecular weight ∼80 kDa in its zymogen form (Ivanov *et al.*, 2017 ▸). Proteolytic activation results in a noncatalytic heavy chain (HC) of 50 kDa connected via a disulfide bridge to a light chain containing the protease domain (Pathak *et al.*, 2019 ▸). FXII contains a fibronectin type II domain (FnII), epidermal growth factor-like domains (EGF1 and EGF2), a fibronectin type I domain (FnI), a kringle domain, a proline-rich region (PRR) and a C-terminal protease domain (Fig. 1 ▸*b*). A missense mutation in the human *F12* gene kringle domain results in FXII with a Trp268Arg change that is linked to cold-induced urticarial auto-inflammatory syndrome (Scheffel *et al.*, 2020 ▸), while Thr309Lys in the PRR is linked to the disorder hereditary angioedema (Cichon *et al.*, 2006 ▸).

The interaction of FXII with polyanions is mediated by anion-binding exosites (ABEs) located in the N-terminal domains. FXII also has Zn^2+^-binding sites located in the EGF1 and FnII domains (Heestermans *et al.*, 2021 ▸). The FnII and kringle domains have been speculated to be important for the regulation of FXII conformational changes (Clark *et al.*, 2020 ▸; Hofman *et al.*, 2020 ▸; Kaira *et al.*, 2020 ▸). To investigate FXII ligand binding, we determined the crystal structures of the five N-terminal domains of FXII (FXII^HC5^) and of the FnII domain (Supplementary Fig. S1) bound to the cofactor Zn^2+^.

## 2. Materials and methods

### 2.1. FXII protein expression and purification

DNA constructs spanning the FnII–EGF1–FnI–EGF2–kringle domains (termed FXII^HC5^) and FnII domain (termed FXII^FNII^) were generated by PCR amplification from the *FXII* gene and ligated into the pMT-PURO vector using BglII and MluI restriction (Supplementary Fig. S1*a*). The FXII^HC5^ and FnII constructs were expressed using *Drosophila* Schneider 2 (S2) cells. For each construct, transfections were performed at a cell density of 1 × 10^6^ cells ml^−1^ of S2 cells in 5 ml serum-free Schneider medium (Invitrogen) supplemented with 10% heat-inactivated fetal bovine serum (FBS) at 28°C using calcium phosphate and grown for 24 h prior to selection with 10 µg ml^−1^ puromycin (Sigma) to establish stable cell lines. After the selection of stable transformants, for the expression of recombinant proteins the S2 culture medium was gradually replaced with Express Five medium without FBS. After the secretory expression of recombinant proteins, the media were collected and centrifuged to remove cell debris and filtered using a 0.22 µm filter. For the purification of FnII, the resultant sample was diluted with an equal volume of 0.025 *M* MES pH 6.0, applied onto a Capto-S column (Cytiva) and eluted using a gradient of 0–1.0 *M* NaCl. Subsequently, the sample was applied onto a HisTrap column (Cytiva) and eluted using a gradient of 0–0.5 *M* imidazole, followed by a final purification step utilizing gel filtration on a HiLoad Superdex 75 16/60 preparatory-grade column equilibrated with 0.05 *M* Tris–HCl pH 8.0, 0.1 *M* NaCl. FXII^HC5^ was captured from the medium using an ion-exchange Capto-S column (Cytiva) equilibrated with 0.02 *M* Na HEPES pH 7.0; a gradient of 0–1.0 *M* NaCl was used for elution. The fractions were pooled, applied onto a HisTrap column and eluted using a 0–0.5 *M* imidazole gradient. Finally, FXII^HC5^ was purified on a HiLoad Superdex 200 16/60 column in 0.05 *M* Tris–HCl pH 8.0, 0.2 *M* NaCl. FXII^HC5^ yields were in the range of 5–10 mg pure protein per litre of insect-cell medium. Lastly, a FXII plasmid construct for recombinant FXII^FnII–EGF1^ (residues 1–112) was also prepared, expressed and purified in insect-cell medium using the method described above (unpublished data).

### 2.2. Crystallization, data collection and structure solution of FXII^HC5^

Crystallization of FXII^HC5^ in 20 m*M* Na HEPES pH 7.0 was carried out in sitting-drop MRC plates using a Mosquito (TTP LabTech) and commercial protein crystallization screens from both Molecular Dimensions and Hampton Research. FXII^HC5^ was concentrated to 8 mg ml^−1^. Small crystals were observed using condition A8 of the MIDASplus screen (Molecular Dimensions) containing 5% polyacrylic acid sodium salt (PAA) 2100. The crystallization condition was optimized by using PAA polymers with varying molecular weights of 1200, 2100, 5100, 8000 and 15 000 using a grid of concentrations from 3.8% to 6%. In addition, additive screens from both Molecular Dimensions and Hampton Research were utilized to improve the crystal size. The final data set was collected from a single large crystal (0.3 × 0.3 × 0.1 µm) grown in a sitting drop with 2 µl protein solution added to 2 µl of a reservoir solution consisting of 5% PAA1200 with the addition of 0.2 µl Hampton Research Additive Screen Reagent D8 containing 0.1 *M* urea.

Data collection was performed on beamline I03 at Diamond Light Source to a resolution of 3.4 Å and data were processed using the beamline implementation of *autoPROC* and *STARANISO* for reduction in space group *I*2_1_2_1_2_1_. Molecular replacement with *Phaser* (McCoy *et al.*, 2007 ▸) used the crystal structures of FXII FnII and FnI–EGF2 (PDB entry 4bdw; Beringer & Kroon-Batenburg, 2013 ▸) and a homology model of the kringle domain prepared using *AlphaFold* (Jumper *et al.*, 2021 ▸) as templates. Additional density was observed for the FnII domain in only two copies of the FXII^HC5^ structure. Manual model building was performed in *Coot* (Emsley *et al.*, 2010 ▸) and structure refinement was performed using *Phenix* (Liebschner *et al.*, 2019 ▸). Evaluation of the quality of the final model was carried out using *MolProbity* (Table 1 ▸; Chen *et al.*, 2010 ▸). The final FXII^HC5^ electron density is continuous for the main chain, spanning residues 18–278, in two molecules, while the FnII domain is absent in a third molecule (residues 77–278; Supplementary Fig. S4).

### 2.3. Crystallization, data collection and structure solution of FXII^FnII^

Sparse-matrix screening with recombinant FXII^FnII–EGF1^ and FXII^FnII^ proteins only resulted in crystals of the FnII domain. Equal volumes of 6 mg ml^−1^ FXII^FnII^ and reservoir solution consisting of 0.01 *M* zinc chloride, 0.1 *M* sodium acetate pH 5.0, 20%(*w*/*v*) PEG 6000 were mixed in a sitting-drop plate and incubated at 19°C. Single crystals grew overnight and were soaked in cryoprotectant comprised of the reservoir supplemented with 30%(*v*/*v*) glycerol and subsequently flash-cooled in liquid nitrogen. Data collection for FXII^FnII^ was carried out on beamline ID23-2 at the ESRF synchrotron and a complete data set was collected to 1.2 Å resolution from a single crystal. Data were indexed and integrated using *MOSFLM* (Battye *et al.*, 2011 ▸). The initial phases were determined with *Phaser* using a previous crystal structure of FXII^FnII^ bound to gC1qR (PDB entry 6szw; Kaira *et al.*, 2020 ▸). Model building was carried out using *Coot*, and *REFMAC*5 and *Phenix* (Murshudov *et al.*, 2011 ▸; Liebschner *et al.*, 2019 ▸) were used for refinement. The N-terminal 20 amino acids of FXII are not observed in the electron density and are presumed to be flexible. The refined models were validated by *MolProbity* (Chen *et al.*, 2010 ▸), and *PyMOL* (https://www.pymol.org) was used to produce figures. The FXII^HC5^ and FXII^FnII^–Zn^2+^ complex crystal structures have been deposited in the PDB (https://www.rcsb.org) with accession codes 8os5 and 7prj, respectively.

### 2.4. In-solution deglycosylation of FXII^HC5^ under native conditions

PNGase F was used for the digestion of recombinant human FXII^HC5^ according to the protocol for nondenaturing reaction conditions provided by the supplier (New England Biolabs, Catalogue No. P0708S). The reaction was set up in the absence of detergent or denaturants, using a tenfold higher enzyme:protein ratio than recommended for denaturing conditions. The reaction was incubated for 6 h at 37°C and subsequently analysed by SDS–PAGE.

### 2.5. Mass spectrometry (MS) of FXII^HC5^

MS studies were performed at the University of York MS facility. For denaturing MS, FXII^HC5^ protein was diluted 1:20 into aqueous 50% acetonitrile containing 1% formic acid. For native-mode mass spectrometry, the protein was buffer-exchanged using Amicon MW spin filters into 1 *M* ammonium acetate pH 7.0. The protein solution was infused at 3 ml min^−1^ into a Bruker maXis qTOF mass spectrometer via an electrospray ionization source. Source conditions and ion-optic parameters were adjusted to favour the detection of native protein states, specifically: dry gas, 250°C at 6 l s^−1^; funnel RF, 400 Vpp; multipole RF, 200 Vpp; quadrupole low cutoff, 1200 *m*/*z*; quadrupole ion energy offset, 3 eV; prepulse storage, 50 ms; transfer time, 160 ms. Spectra were summed over 1 min acquisitions at 0.1 Hz. The data were smoothed (0.2 Da, one cycle, Gauss) by maximum-entropy deconvolution to average masses at 200 Da resolution. Separate deconvolutions were performed for the charge-state regions resulting from the protein monomer and dimer and in total cover the mass range 20–80 kDa. Data acquisition was performed using Bruker *Hystar* and *oTof control* (version 4.1). Peak picking and spectral processing were undertaken using the Bruker *Data Analysis* software with MaxEnt deconvolution (version 4.4).

### 2.6. Small-angle X-ray scattering (SAXS) measurements and analysis

SAXS experiments were carried out on beamline B21 at Diamond Light Source, Didcot, UK using a monochromatic X-ray beam (λ = 0.9408 Å) at an electron energy of 13.2 keV. The beamline was equipped with an EIGER X 4M detector (Dectris, Switzerland) and the sample-to-detector distance was 3.712 m, covering a momentum-transfer range of 0.0045 < *q* < 0.34 Å^−1^, where *q* = (4πsinθ)/λ and 2θ is the scattering angle. In-line gel-filtration-coupled SAXS for human FXII^HC5^ at ∼1.5 mg ml^−1^ was carried out by loading 45 µl protein sample onto a 2.4 ml Superose 6 column (GE Healthcare) equilibrated with a buffer solution consisting of 20 m*M* Tris–HCl pH 8.0, 150 m*M* NaCl and coupled to an Agilent 1200 HPLC system with a flow rate of 0.15 ml min^−1^ at a controlled temperature of 15°C. 600 SAXS data frames were collected with exposure intervals of 3 s and the buffer frames used for the background-subtracted SAXS were collected after 1.5 column volumes. Raw SAXS 2D images were processed with the *DAWN* package, the processing pipeline available at the beamline, to produce normalized, integrated 1D unsubtracted SAXS curves. Initially, the scattering image frames were spherically averaged, scaled and merged using the in-house software *ScÅtter* version 4.0. The radius of gyration (*R*_g_) was estimated through the Guinier approximation, *I*(*q*) = 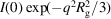, *qR*_g_ < 1.3, and by using *ScÅtter*; the same was performed for the pair distribution of the particle, *p*(*r*), and the maximum dimension *D*_max_, which also were computed using *ScÅtter*. The *BayesApp* (Hansen, 2012 ▸) program was used to further fine-tune the pair distribution *p*(*r*), together with Guinier analysis, the Porod plot and the Kratky plot generated by Bayesian indirect Fourier transformation (BIFT; Vestergaard & Hansen, 2006 ▸). The *SAXSMoW* server was utilized to calculate the molecular weight (de Oliveira Neto *et al.*, 2022 ▸; Piiadov *et al.*, 2019 ▸). The rescaled data with potential outliers removed after error assessment (Larsen & Pedersen, 2021 ▸) were input to *DENSS* (version 1.6.3) to reconstruct 20 *ab initio* 3D electron-density maps from the 1D solution scattering profile and to perform alignment and averaging of the reconstructions (Grant, 2023 ▸). The 3D MRC-formatted density maps were visualized using the *UCSF Chimera* graphical software (Meng *et al.*, 2023 ▸) and to fit the FXII^HC5^ crystal structure. A summary of the SAXS data collection and processing is given in Table 2 ▸. Based on the FXII^HC5^ structure, the X-ray scattering profiles were computed and the discrepancies between the experimental and theoretical SAXS curves were quantified by the minimized χ parameter using the *FoXS*/*MultiFoXS* web server (Schneidman-Duhovny *et al.*, 2016 ▸).

### 2.7. Gel-filtration analysis of FXII in the presence of polyanions

The complex of FXII in the presence of heparin and polyP was analysed by analytical size-exclusion chromatography (gel filtration) using an ÄKTAmicro FPLC system (Cytiva, Little Chalfont, UK). Narrowly size-fractionated polyP preparations were produced from chemically synthesized polyP using preparative PAGE performed on a Model 491 Prep Cell (Bio-Rad) using a 100 ml gel and a 30 mg polyP load. PolyP concentrations were quantified by measuring inorganic phosphate after complete hydrolysis, as described by Smith *et al.* (2018 ▸). Preparations collected from fractions were sized as described previously (Smith *et al.*, 2018 ▸) and are referred to by their polymer lengths, which were 34, 55 and 69 phosphate units. PolyP was added to FXII at different molar ratios from 1:1 to 1:100 with plasma-purified FXII (Enzyme Research Laboratories). Gel filtration was performed on the resulting mixture using a 3 ml Superose S200 column (Cytiva) equilibrated with 0.05 *M* HEPES–HCl pH 7.0, 0.2 *M* NaCl at a flow rate of 0.3 ml min^−1^. Heparin fragment Hep20 was purchased from Iduron (termed dp20) and was added to FXII at different molar ratios from 1:1 to 1:10 and gel filtration was performed as described for polyP. For FXII^HC5^ gel-filtration studies with heparin, an ÄKTApure FPLC system (Cytiva, Little Chalfont, UK) was used. Heparin was added to FXII at different molar ratios from 1:5 to 1:10 and gel filtration was performed as described for polyP. The column was calibrated with standard proteins (Cytiva, Little Chalfont, UK) of known Stokes radii: thyroglobulin (669 kDa), ferritin (440 kDa), aldolase (158 kDa), conalbumin (75 kDa), carbonic anhydrase (29 kDa) and aprotinin (6.5 kDa). Blue dextran MW 2000 kDa was used to determine the column void volume (*V*_o_). Elution volume positions were monitored at 280 nm. The partition coefficient (*K*_av_) of FXII and protein standards was calculated using the equation *K*_av_ = (*V*_e_ − *V*_o_)/(*V*_t_ − *V*_o_), where *V*_e_ is the elution volume and *V*_t_ is the total column volume.

## 3. Results

### 3.1. Crystal structure of FXII^HC5^

Constructs spanning different combinations of the FXII N-terminal domains were expressed using insect cells and purified from media as described in Section 2 (Supplementary Fig. S1). FXII^HC5^ yields were in the range of 5–10 mg pure protein per litre of insect-cell medium (Supplementary Fig. S1) and the amino-acid sequence was confirmed using mass spectrometry (MS; Supplementary Fig. S2). Data were collected to 3.4 Å resolution from a single crystal (Table 1 ▸) and the FXII^HC5^ structure was solved by molecular replacement using the available crystal structures of the FXII FnII (Kaira *et al.*, 2020 ▸) and FnI–EGF2 domains (Beringer & Kroon-Batenburg, 2013 ▸). The FXII^HC5^ structure as viewed in Fig. 1 ▸(*c*) has a circular ring or torc shape, with the kringle domain forming a head-to-tail contact via a latch-like loop from the FnII domain (Fig. 1 ▸*d*). The FnII domain is followed by a nine-residue linker loop spanning residues 70–78, which terminates in a sharp 90° angle leading into the tandem arrangement of the EGF1–FnI domains. The structure was analysed for stable interfaces with *PDBePISA* (Krissinel & Henrick, 2007 ▸), which identified that the FXII^HC5^ monomers interlock in an antiparallel arrangement, which buries a surface area of 2067 Å^2^. This FXII^HC5^ dimer structure positions the FnII domains radially 75 Å apart, whereas the two kringle domains are located axially 20 Å apart (Figs. 1 ▸*e* and 1 ▸*f*).

**Figure 2:**
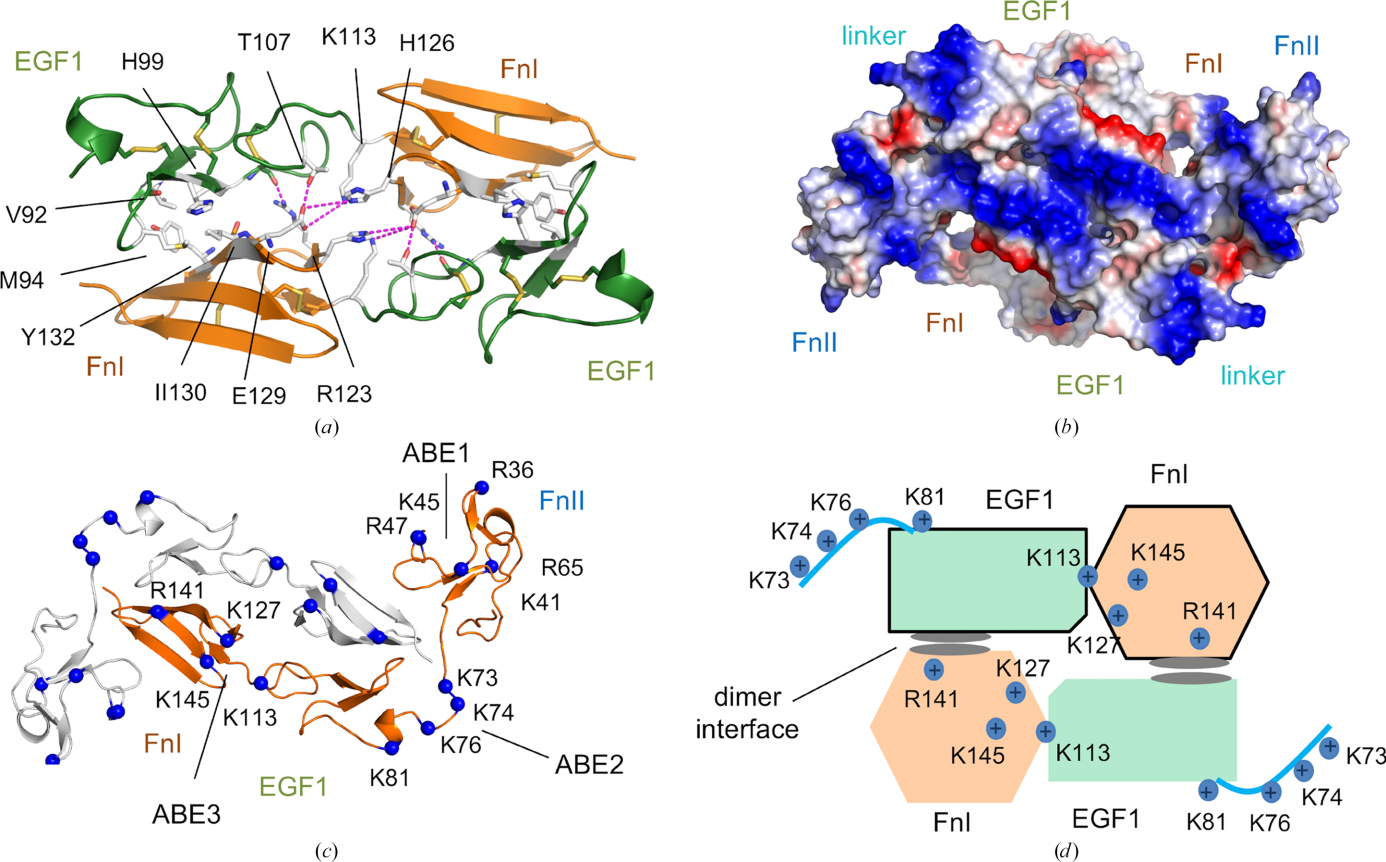
FXII^HC5^ intramolecular interactions and charge distribution. (*a*) Dimer interfacial interactions between the EGF1 and FnI domains are shown with key residues as sticks and electrostatic bonding as purple dotted lines. (*b*) A charged molecular-surface representation (blue, positive; red, negative) with a continuous flat surface of positive charge generated centrally by the EGF1–FnI domain dimer. Two radial surfaces of further positive charge are generated by the linker and FnII domains. (*c*) Cartoon diagram showing the dimer with the two L-shaped FnII–EGF1–FnI polypeptides. Clusters of surface-exposed arginine or lysine residues are shown as sticks coloured blue and labelled as anion-binding exosites (ABE). (*d*) A schematic diagram illustrates the EGF1 and FnI domains with the location of charged residues for ABE2 and ABE3 indicated in the context of the dimer interface (grey).

In the asymmetric unit there are three copies of FXII^HC5^. Two FXII^HC5^ monomers are observed with the FnII domain and linker region present. In one FXII^HC5^ monomer the FnII domain is absent and the EGF1–FnI–EGF2–kringle domains exhibit a slightly different conformation and a reduced buried surface area of 1408 Å^2^. The linker-loop residues can be thought of as a flexible strap enabling the FnII domain to wrap around the FnI domain and interlock in a tight-knit arrangement contacting the kringle domain (Supplementary Movie S1). The antiparallel EGF1–FnI domain interface is formed from residues 103–107 and 123–129, with electrostatic interactions in the centre flanked by hydrophobic contacts on either side (Fig. 2 ▸*a*). Hydrogen bonds are formed between the Arg123 side chain and the main-chain carbonyl of Cys102, and the Glu129 carboxyl forms a hydrogen bond to the hydroxyl side chain of Thr107. Additional electrostatic interactions occur between Glu129 and His126 and Lys113.

Biochemical studies have previously identified contributions to polyanion binding from the FnII, EGF1 and FnI domains (Shamanaev *et al.*, 2022 ▸). A distinctive feature of our FXII^HC5^ dimer structure is the symmetric presentation of three areas of positively charged residues [termed the anion-binding site (ABE); Figs. 2 ▸*b* and 2 ▸*c*]. The FnII domain arranges His35, Arg36, His40, Lys41, His44 and Lys45 into ABE1, which is a triangular-shaped flat surface (Supplementary Movie S2). The linker-region residues Lys73, Lys74 and Lys76 cluster with His78, Lys81 and His82 in the EGF1 domain to generate an area of positive charge, and these residues have been identified as the polyP-binding site (ABE2; Shamanaev *et al.*, 2023 ▸). A third cluster of basic residues, Lys113, Lys127, Lys145 and Arg141, centred around the FnI domain results in an extended pocket of symmetric positive charge close to the dimer axis (ABE3; Fig. 2 ▸*d*). The FXII^HC5^ dimer that we observe may not occur in full-length FXII due to steric and structural constraints imposed by the presence of the C-terminal PRR and protease domain.

### 3.2. The FXII^HC5^ kringle domain contains a lysine-binding site

**Figure 3:**
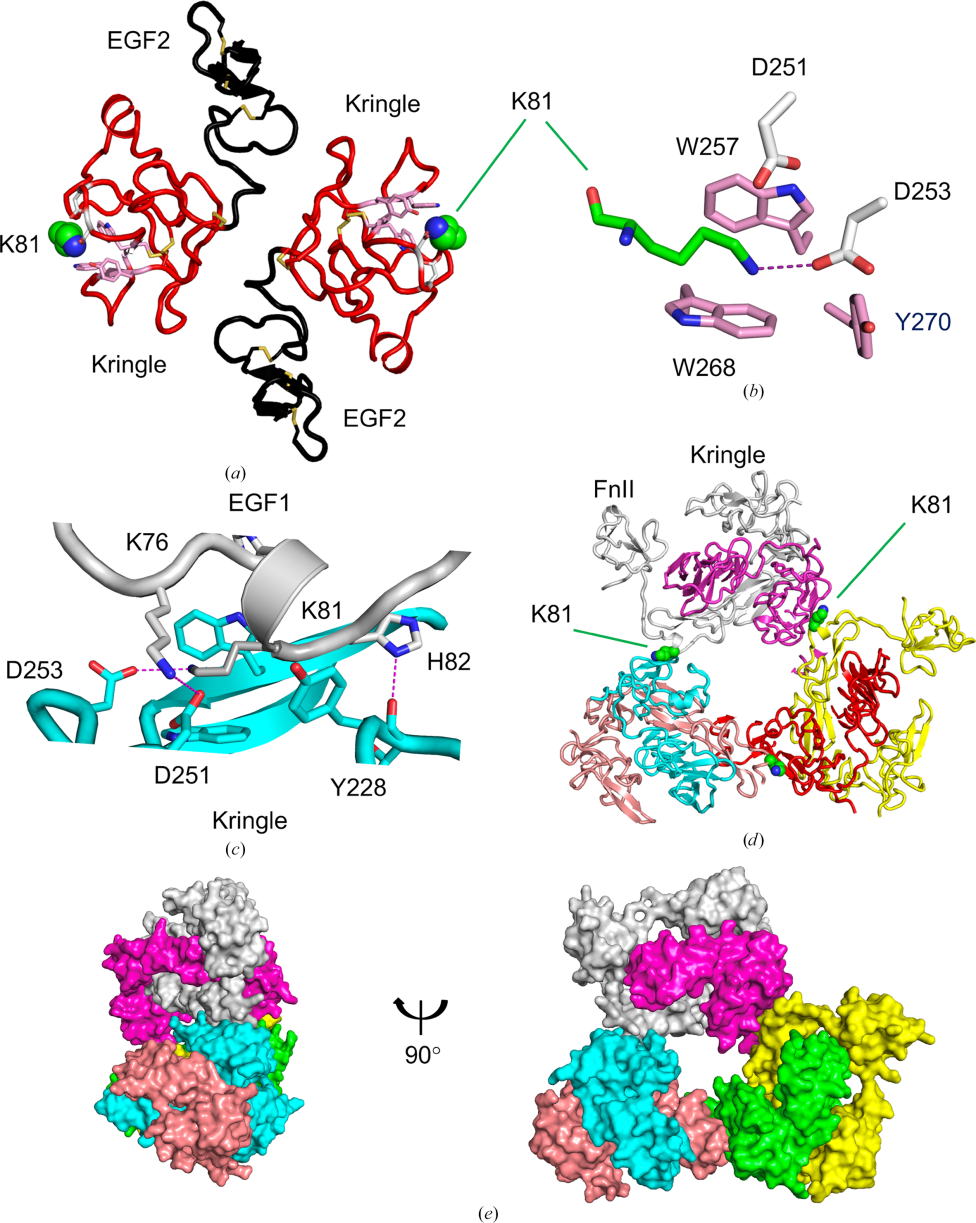
FXII^HC5^ kringle lysine-binding site. (*a*) Cartoon diagram of FXII^HC5^ in the region of the EGF2 (black) and kringle (red) domains showing the outward-facing lysine-binding sites as sticks engaging the Lys81 side chain (green spheres). (*b*) Enlarged view of EGF1 Lys81 (green) and the kringle domain lysine-binding site residues shown as sticks. (*c*) The interface between the EGF1 and the kringle domains involves positive charges on Lys76, Lys81 and His82 from the EGF1 domain coordinating to the kringle domain residues. Electrostatic interactions are shown as purple dotted lines. (*d*) Kringle-mediated interactions result in three FXII^HC5^ dimer forms being arranged into a cyclic hexamer. This hexameric form of the FXII^HC5^ structure is represented as a ribbon with the subunits coloured purple, grey, green and yellow and the FnII-less FXII^HC5^ dimer coloured cyan and salmon. (*e*) Two views of the hexameric form of FXII^HC5^ shown as a molecular surface.

The FXII kringle domain contains a lysine-binding site, with a groove formed by Trp257 and Trp268 flanked by acidic residues Asp251 and Asp253 that interacts with Lys81 from EGF1 on an adjacent FXII^HC5^ structure (Figs. 3 ▸*a* and 3 ▸*b*). In the FXII^HC5^ structure two kringle domains are arranged such that the lysine-binding sites point outwards separated by a distance of 50 Å (Fig. 3 ▸*a*). This is a characteristic orientation, with the extended lysine alkyl chain interacting with the Trp257 and Trp268 side chains and the amine forming electrostatic interactions with the side chains of Asp251 and Asp253. The FXII kringle domain lysine-binding site bears a close resemblance to the tissue-type plasminogen activator (tPA) kringle crystal structure (de Vos *et al.*, 1992 ▸) and the tandem EGF2–kringle inter-domain angle of 90° has been observed previously in the crystal structure of the amino-terminal domains of the urokinase plasminogen activator (uPA; Barinka *et al.*, 2006 ▸). Compared with the plasminogen kringle domain 1 structure, the FXII kringle domain does not have the required basic residues (plasminogen kringle 1 Lys35 and Arg71) for terminal lysine binding (Mathews *et al.*, 1996 ▸).

The FXII^HC5^ kringle–EGF1 interaction resembles a crystal contact and buries a small surface area whereby EGF1 α-helix residues Lys76 and Lys81 interact with the kringle Asp251 and Asp253 side chains (Fig. 3 ▸*c*). Additional interactions are formed by the packing of the Tyr228 and Lys81 side chains, and the Tyr228 main-chain carbonyl hydrogen-bonds to the side chain of His82. These intermolecular FXII^HC5^ inter­actions involving the EGF1–kringle domains assembles three FXII^HC5^ dimers into a doughnut-shaped hexamer (Figs. 3 ▸*d* and 3 ▸*e*).

### 3.3. MS analyses of recombinant FXII^HC5^

**Figure 4:**
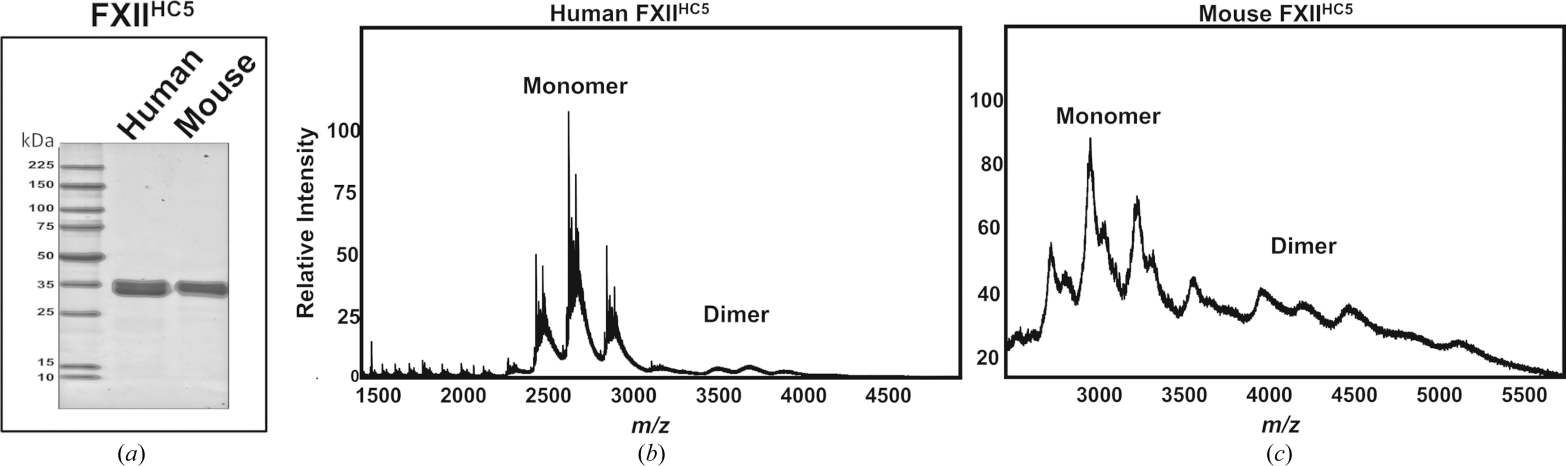
Native mass spectrum of human FXII^HC5^. (*a*) Coomassie-stained SDS–PAGE gel for purified recombinant human and mouse FXII^HC5^. (*b*, *c*) Native mass spectrum of (*b*) human FXII^HC5^ and (*c*) mouse FXII^HC5^ showing multiple charge-state distributions; different species are labelled as monomer and dimer peaks.

Analysis of the interfaces in the FXII^HC5^ crystal structure using *PDBePISA* (Krissinel & Henrick, 2007 ▸) indicates that the large surface area buried by the structure shown in Fig. 1 ▸(*f*) should be stable as a dimer. We thus utilized MS (Rostom & Robinson, 1999 ▸; Karch *et al.*, 2022 ▸) to probe whether a FXII^HC5^ dimer could be detected in the gas phase. To gain a precise measurement of the mass, we first used denaturing MS, revealing a major peak for FXII^HC5^ at ∼34.8543 kDa (Supplementary Fig. S3). In the presence of PNGase F a shift in mass indicated the removal of a single N-glycan (Supplementary Fig. S3). Next, native MS was used to analyse oligomer formation of FXII^HC5^ (Supplementary Fig. S3) and showed both charged-state and deconvoluted spectra and detected a monomer peak at 34.9 kDa and a small dimer peak at a molecular weight of 67.1 kDa. We next purified mouse FXII^HC5^ for comparison, which has ∼70% sequence identity to human FXII^HC5^. Mouse FXII^HC5^ also revealed a monomer peak of 35.9 kDa and a small dimer peak that is precisely double this at 71.9 kDa (Fig. 4 ▸ and Supplementary Fig. S3). In data acquisition under native-favouring conditions there is a shift in the charge-state distribution towards a lower charge state and range (higher *m*/*z*), which is typical of native structures that are more folded and so have less accessibility for protonation. Overall, these analyses revealed that the signal is dominantly native low-charge monomer and relatively less poorly resolved low-charge dimer, as annotated in the spectrum obtained. Although protein multimerization can be measured by MS in the gas phase, this is very much protein-dependent.

### 3.4. SAXS characterization of FXII^HC5^

**Figure 5:**
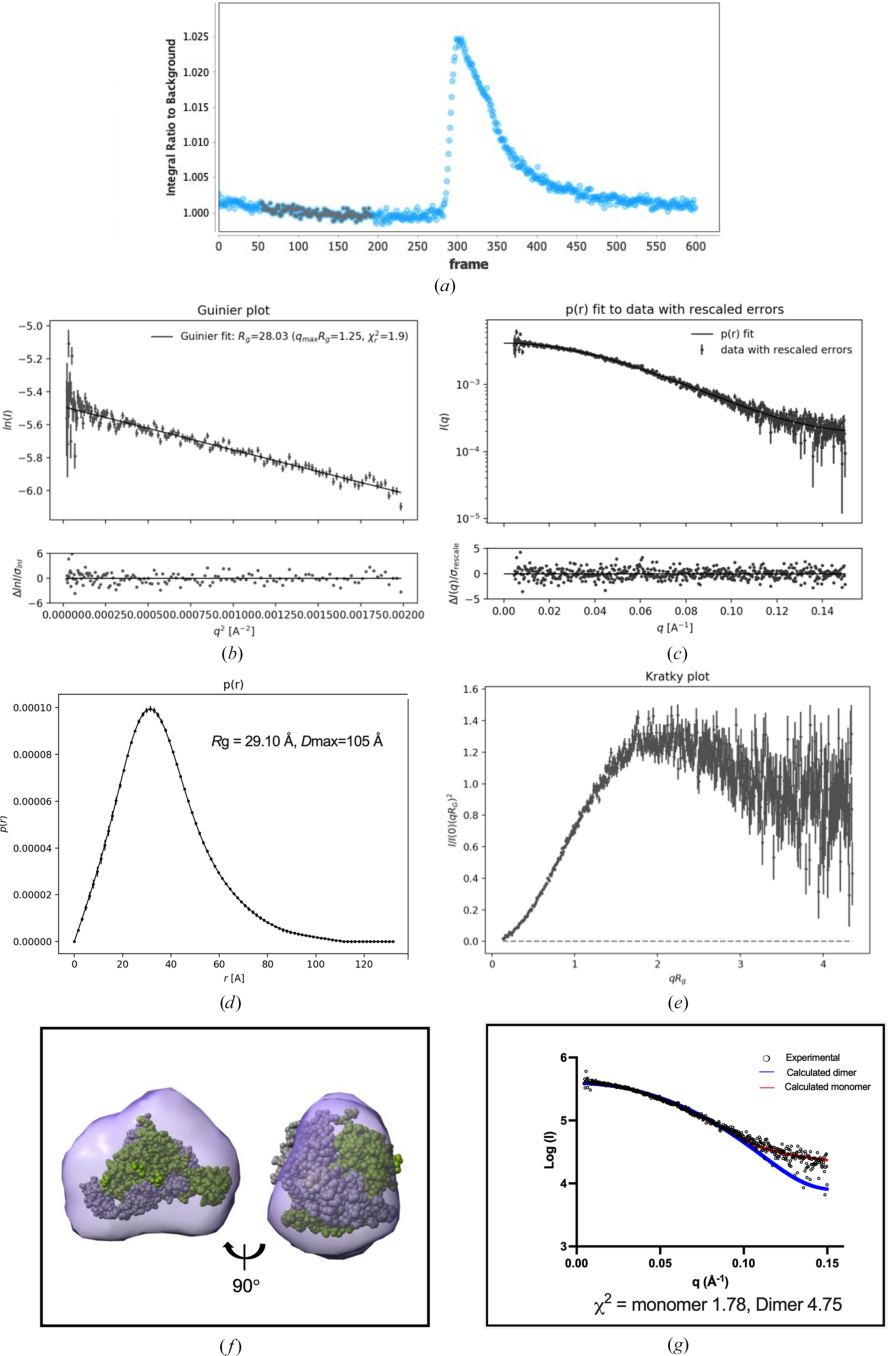
SAXS analysis of human FXII^HC5^. (*a*) SEC-SAXS signal plot. Each point represents the integrated area of the ratio of the sample SAXS curve to the estimated background. The frames used as the buffer background are in grey with the average represented as a grey horizontal line. (*b*) Guinier plot. (*c*) Distance distribution function fit to the data with rescaled errors. (*d*) Distance distribution. (*e*) Kratky plot. The plots in (*b*)–(*e*) are taken from *BayesApp*. Fit of the interlocking dimeric crystal structure (*f*) into the 32 Å density envelope obtained from *DENSS*. (*g*) Comparison of the experimental SAXS curve (circles) with the curve calculated from the crystal monomeric (red) and dimeric (blue) structure models for data covering a momentum-transfer range of 0.0045 < *q* < 0.15 Å^−1^. The goodness-of-fit parameter from *FoXS* (χ^2^) is 1.78 for the monomer, with *R*_g_ = 25.54 Å, *c*_1_ = 0.99 and *c*_2_ = 3.23, and 4.75 for the dimer, with *R*_g_ = 26.95 Å, *c*_1_ = 1.05 and *c*_2_ = −2.00.

We next used SAXS to investigate whether FXII^HC5^ dimer formation could be detected in solution. A single broad peak was observed during gel filtration (Fig. 5 ▸*a*) and the resulting SAXS data were of high quality, with no evidence of aggregation from the Guinier plot (Fig. 5 ▸*b*). The radius of gyration (*R*_g_) values from both Guinier and Porod analysis are in good agreement at 28.03 and 29.10 Å, respectively, with a maximum particle dimension (*D*_max_) of 105 Å (Figs. 5 ▸*c* and 5 ▸*d*, Table 2 ▸). This compares favourably with the calculated FXII^HC5^ dimer *R*_g_ of 26.95 and longest dimension of 96 Å. The smaller value of the calculated *R*_g_ compared with the experimental value may be explained by hydration and the flexible N-terminus (residues 1–17) and C-terminus (residues 278–295), which are not defined in the crystal structure. The molecular-weight estimate calculated via *SAXSMoW* is 51 kDa (Piiadov *et al.*, 2019 ▸; de Oliveira Neto *et al.*, 2022 ▸).

Fig. 5 ▸(*f*) shows two views of the SAXS-derived electron-density map (∼32 Å resolution). The left-hand view showing the profile is triangular in shape and the interlocking FXII^HC5^ dimeric model from the crystal structure can be comfortably fitted into the 3D reconstruction (Fig. 5 ▸*f*). The right-hand view in Fig. 5 ▸(*f*) also shows that the dimensions match the model, but the narrowing of the density in the middle may represent an average of both monomer and dimer. To summarize, three-dimensional electron-density reconstruction using *DENSS*, which does not strictly rely on assumptions of globularity, yielded a volume consistent with an average particle size indicative of both monomeric and dimeric forms. The fitted electron-density map corroborates the dimensions and spatial arrangement consistent with the interlocking dimer in the crystal structure, reinforcing the presence of dimeric forms in solution. The SAXS data were further analysed by calculating theoretical SAXS data from the crystal structure using *FoXS*/*MultiFoXS*. Comparison of the experimental SAXS curve (circles) with the curve calculated from the monomeric (red) and dimeric (blue) structure models for data covering a momentum-transfer range of 0.0045 < *q* < 0.15 Å^−1^ is shown in Fig. 5( ▸*g*). The goodness-of-fit parameter from *FoXS* (χ^2^) is 1.78 for the monomer and 4.75 for the dimer, respectively (Fig. 5 ▸*g*).

### 3.5. Gel filtration of FXII with polyanions of different lengths

**Figure 6:**
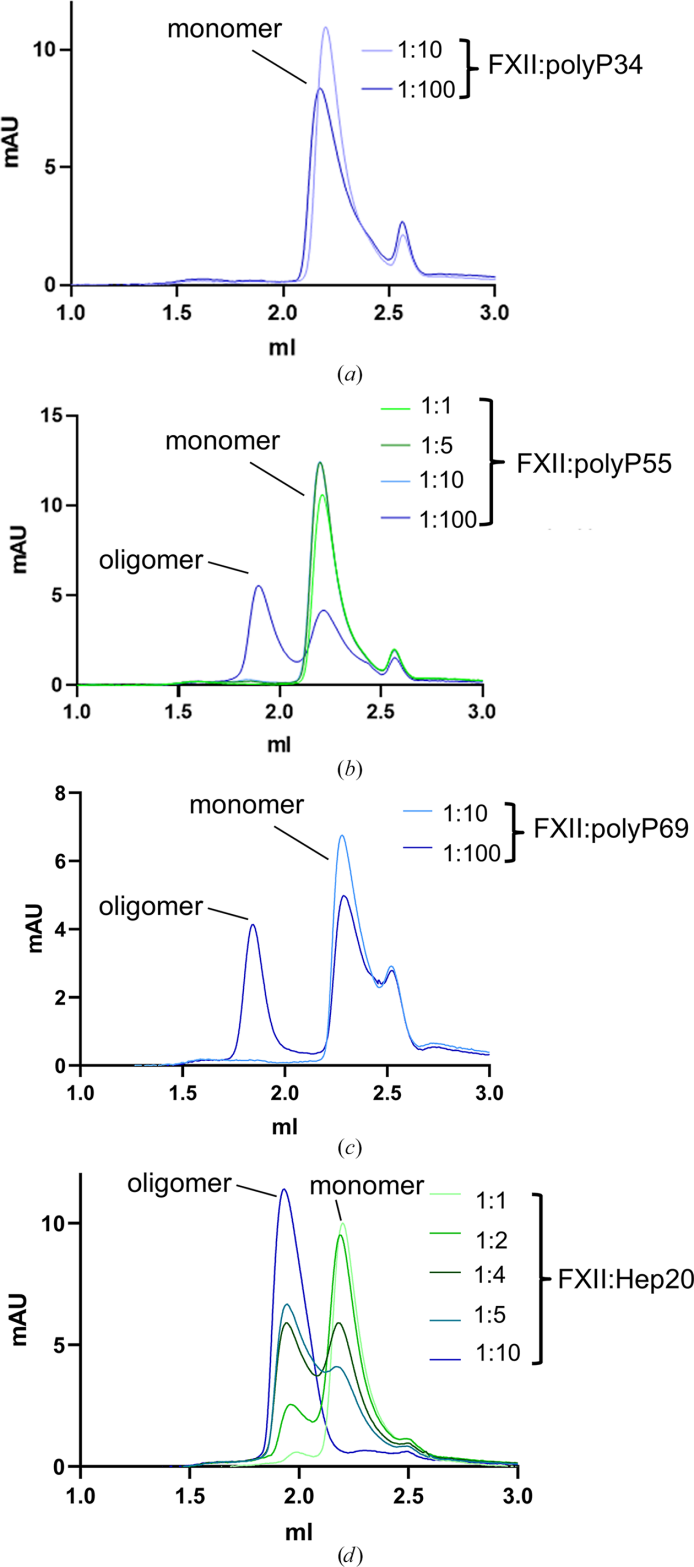
Gel filtration of FXII complexes with polyP and heparin fragments. (*a*, *b*, *c*) Gel-filtration elution profiles for full-length plasma-purified FXII in the presence of increasing molar ratios of polyP with different chain lengths of (*a*) 34, (*b*) 55 and (*c*) 69. Elution volume (*V*_e_) measured in millilitres is shown on the *x* axis and UV absorption (mAU) is shown on the *y* axis. (*d*) Gel-filtration elution profiles for FXII in the presence of increasing molar ratios of heparin fragment Hep20. The second peak arising at a *V*_e_ of 1.92 ml is equivalent to the standard gel-filtration standard ferritin, which has a molecular weight of 440 kDa.

The full-length FXII zymogen is monomeric (Colman *et al.*, 1997 ▸; Kaira *et al.*, 2020 ▸), but oligomerization has been reported to occur in solution in the presence of soluble polyanions (Samuel *et al.*, 1992 ▸; Wang *et al.*, 2019 ▸). We utilized fractionated polyP in a series of gel-filtration experiments to characterize FXII oligomerization at increasing polyP concentrations and chain lengths of 34, 55 and 69 phosphate units. Gel filtration was performed with a Cytiva ÄKTAmicro system and a 3 ml Superose S200 column. The column was calibrated using commercial gel-filtration standards (Cytiva): thyroglobulin (669 kDa), ferritin (440 kDa), aldolase (158 kDa), conalbumin (75 kDa), carbonic anhydrase (44 kDa) and aprotinin (6.5 kDa). The FXII zymogen has an elution volume (*V*_e_) of 2.2 ml and the calculated Stokes radius is 3.2 nm, which is very close to the conalbumin standard (75 kDa), corresponding to an FXII monomer (the calculated molecular weight based on the amino-acid sequence alone is 65 733 Da). Addition of the shortest polymer polyP34 to FXII at increasing concentrations up to a ratio of 1:100 did not result in any difference in *V*_e_ compared with FXII alone (Fig. 6 ▸*a*). In contrast, polyP55 and polyP69 resulted in the appearance of a second peak with a *V*_e_ of 1.92 ml similar to the gel-filtration standard ferritin (1.92 ml) with a molecular weight of 440 kDa.

To extend these FXII oligomerization observations to a second polyanion, we utilized a commercially available (Iduron) fractionated low-molecular-weight heparin composed of ∼20 saccharide units (ten repeats of the main disaccharide unit; MW of ∼5750 Da). A mixture of FXII in the presence of increasing molar ratios of Hep20 resulted in the appearance of second peak at a *V*_e_ of 1.94 ml which became a single peak at a 1:10 ratio excess of Hep20. Hep20 requires a 1:10 ratio to drive FXII oligomer formation and results in a complete conversion to the oligomeric FXII. In contrast, polyP requires a 1:100 molar ratio for the FXII oligomer to appear and the FXII monomer is still present in the elution profile. Interestingly, both the polyanions polyP55 and Hep20, which are of similar molecular weight, give a very similar observed increase in the Stokes radius of the FXII–polyanion complex to 6.2 and 6.1 nm, respectively. The standard ferritin with a molecular weight of 440 kDa has a Stokes radius of 6.2 nm. Additionally, a small subsidiary peak is observed at ∼2.5–2.6 ml; however, this peak corresponds to a significantly smaller estimated size than the FXII domain: ∼7.0–4.0 kDa. This peak did not contain any protein fragment when analysed by SDS–PAGE and may represent potential breakdown products, aggregates or buffer constituents *etc.*, and was treated as irrelevant to the experiment. In these assays, the concentrations of FXII and polyanions were 10 µ*M* and 0.01–1 m*M*, respectively.

### 3.6. FXII Zn^2+^-binding sites

**Figure 7:**
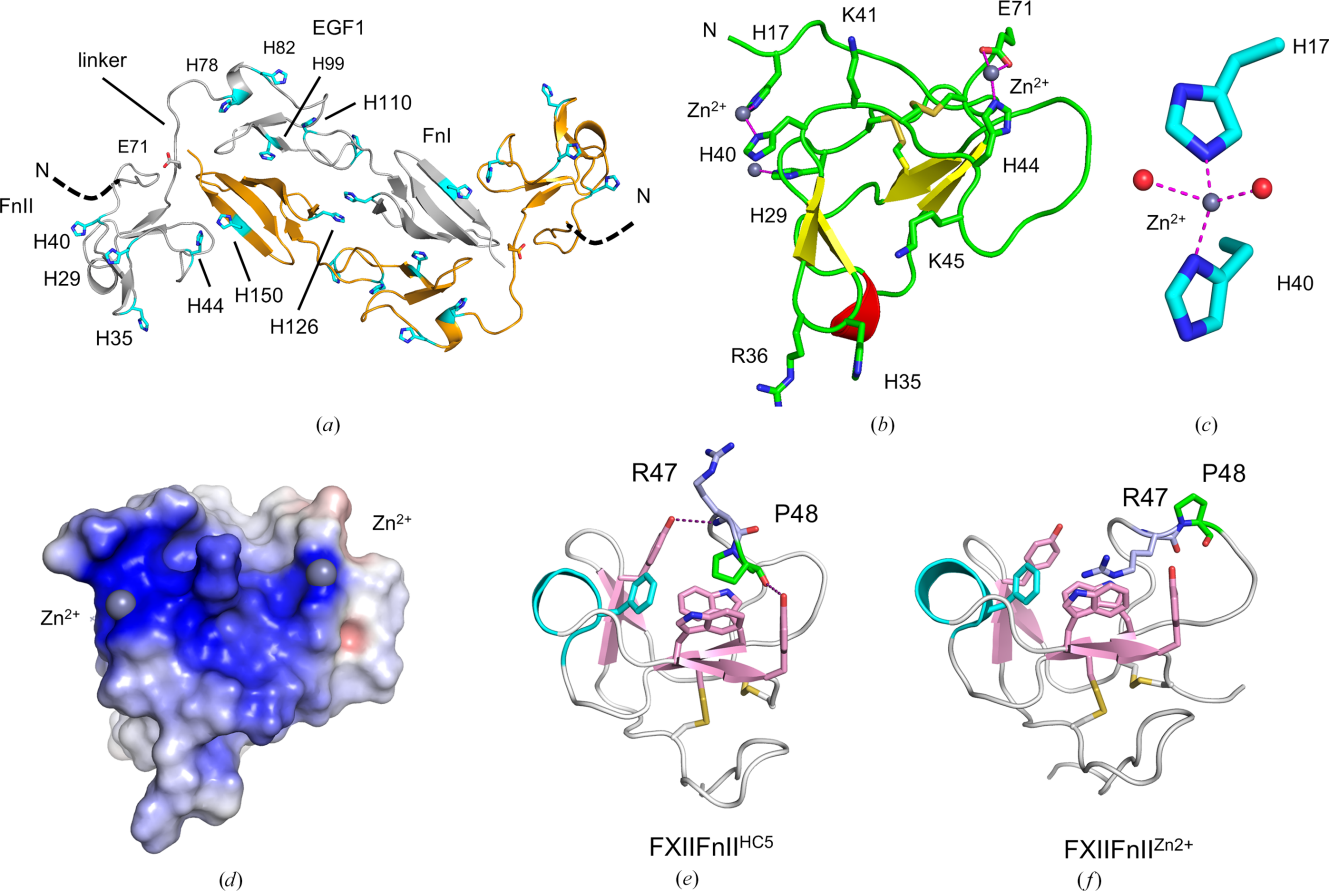
FXII Zn^2+^-binding sites. (*a*) Cartoon diagram of the FXII^HC5^ structure showing the dimer with the two L-shaped FnII–EGF1–FnI polypeptides, with clusters of surface-exposed histidine residues as potential Zn^2+^-binding sites shown as sticks (cyan). The black dashed line labelled N represents the N-terminus. (*b*) Cartoon diagram of the isolated FXII^FnII^ crystal structure with bound Zn^2+^ ions shown as grey spheres. Residue Arg47 is shown in light blue and Pro48 is in green. Other residues are shown as sticks in dark blue. (*c*) Enlarged view of the His17 and His40 residues shown as sticks, with the coordination sphere of the Zn^2+^ ion (grey sphere) completed by two water molecules (red spheres). Purple dotted lines represent electrostatic interactions formed with Zn^2+^. (*d*) A charged molecular-surface representation (blue, positive; red, negative) showing the FXII^FnII^ ABEI domain as a triangular surface of positive charge with two Zn^2+^ ions as grey spheres. Comparison of the FnII domain cation-binding site in the FXII^HC5^ crystal structure, where it is occupied by Pro48 (*e*), compared with the Zn^2+^-bound isolated FXII^FnII^ (*f*), where a loop rearrangement occurs and the the Arg47 guanidinium occupies the cation-binding site.

Biochemical analyses have identified Zn^2+^-binding sites in the FnII and EGF1 domains (Røjkjaer & Schousboe, 1997 ▸) which greatly enhance the enzymatic reactions of FXII (Wang *et al.*, 2019 ▸; Shore *et al.*, 1987 ▸). The precise location of the Zn^2+^-coordinating residues is unknown, but the FXII sequence is histidine-rich (Samuel *et al.*, 1993 ▸) and the FXII^HC5^ structure shows that the FnII–EGF1–FnI domains present ten histidine resides on one contiguous surface (Fig. 7 ▸*a*). A cluster of residues, His78, His82, His99 and His110 in the EGF1 domain and His40, His29, His35 and His44 in the FnII domain, represent potential Zn^2+^ ion coordination sites. To investigate, we expressed untagged FXII FnII domain (residues 1–71, termed FXII^FnII^) and confirmed that the FnII protein did bind to a Zn^2+^ affinity column (unpublished observation). The purified FXII^FnII^ sample was crystallized in the presence of ZnCl_2_ and the structure was determined using molecular replacement (Table 1 ▸). The 1.2 Å resolution FXII^FnII^ structure revealed the expected FnII domain fold of four β-strands arranged as two antiparallel β-sheets, β1–β2 and β3–β4, at right angles, together with a short α-helix (Fig. 7 ▸*b*). Utilizing the 1.2 Å resolution structure factors, we were able to identify two bound Zn^2+^ ions in the anomalous difference Fourier map. Fig. 7 ▸(*b*) illustrates the Zn^2+^ ions as grey spheres; site 1 is coordinated by His17 and His40, with the remaining coordination completed by two water molecules (Fig. 7 ▸*c*); the site 2 Zn^2+^ ion is coordinated by residues His44 and Glu71 with additional coordination from His35 of a symmetry-related molecule. The two Zn^2+^ ions are placed on the same face of the FXII^FnII^ structure as the basic residues Arg36, Lys41 and Lys45, forming a concentrated triangular surface of positive charge (Fig. 7 ▸*d*).

An interesting feature of the FXII^FnII^ Zn^2+^ sites is that they both coordinate residues from the FnII domain flanking sequences. His17 is not present in the FXII^HC5^ structure and residues 1–17 are not visible in the FXII^HC5^ electron density (shown as a dotted line in Fig. 7 ▸*a*), and Glu71 is in the linker region. FnII domains can interact with ligands via a surface pocket formed between residues Trp53 and Trp66 from strands β4–β3 and Phe60 from the α-helix (Morgunova *et al.*, 1999 ▸; Wah *et al.*, 2002 ▸). This FXII^FnII^ pocket is occupied by the Arg47 side chain, which extends to form a cation–π interaction with Trp66. A comparison of FXII^FnII^ with the FXII^HC5^ structure reveals significant conformational change in the FnII latch-loop region. The FXII Arg47-Pro48-Gly49-Pro50 sequence rearranges such that Arg47 ratchets out of the FnII pocket in FXII^HC5^ to form a head-to-tail salt bridge with the kringle domain residue Asp264. In FXII^HC5^ the main-chain carbonyl of Pro48 forms a hydrogen bond to the side-chain hydroxyl of Tyr68 (Figs. 7 ▸*e* and 7 ▸*f*). This 4 Å movement of Arg47 and Pro48 is animated as a molecular morph between the two structures in Supplementary Movie S3.

## 4. Discussion

Human plasma coagulation is mediated by a thrombin-generation mechanism based on vitamin K-dependent proteases which contain the gamma-carboxyglutamic acid-rich (Gla) domain, and the contact system (Ponczek *et al.*, 2020 ▸) based on FXII, PK and FXI. Crystal structures exist for the N-terminal domains of PK and FXI (Li *et al.*, 2019 ▸), revealing a disc-shaped organization of the four apple domains mediated by key interfaces between domains. This allows coordination of the ligand-binding sites in the separate domains (Li *et al.*, 2023 ▸). The domain structure of the first five N-terminal domains of FXII is shared with one other plasma protease, hepatocyte growth factor (HGFA), but there is no structure available to describe how the five N-terminal domains of these proteins assemble and present their respective ligand-binding sites. The FXII^HC5^ structure, as viewed in Fig. 1 ▸(*c*), has a circular ring or torc shape, with the kringle domain forming a head-to-tail contact via a latch-like loop from the FnII domain. Notable features are inter-domain interfaces between the EGF1–FnI–EGF2–kringle domains, but no interface is observed between the FnII and EGF1 domains. Instead, the FnII domain is followed by an extended linker loop spanning residues 70–78 (cyan in Fig. 1 ▸*c*). This nine-residue linker loop and the adjacent EGF1 domain are of interest as two groups have recently identified residues in this region as being critical for polyanion binding (Shamanaev *et al.*, 2022 ▸; Frunt *et al.*, 2024 ▸). The kringle domain-binding site is occupied by a lysine from an intermolecular interaction. The function of lysine binding by the FXII kringle domain is unknown, but it is thought to be involved in an intramolecular interaction that maintains the FXII zymogen in an inactive conformation, as is observed in prothrombin (Chinnaraj *et al.*, 2018 ▸). Two FXII^HC5^ monomers interlock and bury a large surface area. Solution SAXS analysis and native mass spectrometry described the presence of a dimeric form along with the predominant FXII^HC5^ monomer.

We also made observations from the gel-filtration experiments that plasma-purified full-length FXII in the presence of polyP or heparin fragments results in elution volumes consistent with oligomer formation in the presence of polyanions but not for the short-chain polyP34. The recombinant FXII^HC5^ showed similar results to full-length FXII, indicating oligomer formation in the presence of a heparin fragment. These results for polyP and heparin fragments are comparable to a previous study with dextran sulfate, where FXII is visualized as binding on either side of the elongated polyanion (Samuel *et al.*, 1992 ▸). Data with polyP of different lengths also revealed that the short polyP (27–35 units) does not robustly support FXII autoactivation but does result in exposure of the FXII activation loop (Shamanaev *et al.*, 2023 ▸).

Overall, the FXII^HC5^ sequence has a net positive charge, whereas the protease domain is negatively charged. An attractive feature of the FXII^HC5^ dimer structure is that it localizes three distinct clusters of positive charged residues as either flanking ABE1 and ABE2 (FnII and EGF1) or ABE3, centred around the FnI domain, generating a symmetric surface of positive charge which could be utilized for polyanion binding (Figs. 2 ▸*b* and 2 ▸*c*). In terms of contributions to polyanion binding from other domains, a recent study identified residues Gln317–Ser339 from the PRR as being essential for FXII surface binding and activation (Heestermans *et al.*, 2021 ▸). However, a second study using chimeric FXII–PRR and FXII-ΔPRR deletion proteins argues against a critical role for the FXII PRR in surface-dependent autocatalysis (Shamanaev *et al.*, 2022 ▸). The PRR does not contain positively charged residues required for binding polyP, dextran sulfate or heparin, which all have features of repeating negative charge. Data from *in vivo* and *ex vivo* models of cardiovascular disease indicate that FXII-specific antibodies 9A2 and 15H8 targeting the FnI–EGF2–kringle domain region of FXII^HC5^ prevent auto-activation in the presence of polyanions and block thrombus formation (Matafonov *et al.*, 2014 ▸).

Zn^2+^ ions are critical for efficient FXII activation (Wang *et al.*, 2019 ▸). Zn^2+^ is central to the process of platelet-dependent FXIIa generation and plays an important role in thrombus formation *in vivo* (Chaudhry *et al.*, 2020 ▸). The FXII^FnII^ crystal structure that we present here reveals the positions of two surface-bound Zn^2+^ ions. Overall, the FXII^FnII^ structure is similar to the matrix metalloproteinase-9 (MMP9) FnII domain crystal structure, which also has the solvent-exposed hydrophobic pocket formed by Trp53 and Trp66 and a flanking Arg47 (Morgunova *et al.*, 1999 ▸). In the MMP9 structure Arg47 (which is numbered differently in MMP9) is utilized to form an intramolecular interaction with a peptide of sequence FPGD from the C-terminus. Another available ligand complex is the structure of the seminal plasma protein 109 (PDC-109) FnII domain phosphocholine complex (Wah *et al.*, 2002 ▸). Arg47 and Arg368 in FXII FnII and MMP9 FnII, respectively, are not conserved in PDC-109 FnII and are replaced by a serine (Ser88). This change allows the PDC-109 FnII binding pocket to accommodate the bulky phosphate group as a ligand.

An important limitation of this study is that the FXII^HC5^ construct used does not have the FXII PRR and protease domain present and the inter-chain interactions may not be fully representative of native FXII. The inter-chain inter­actions between the FXII^HC5^ monomers that we observe may be contacts that would otherwise be occupied by intramolecular interactions from the C-terminal domains, as is thought to occur in the FXII zymogen. However, when the FXII zymogen becomes activated it is possible that this disrupts these intramolecular interactions, such that in FXIIa the N-terminal domains self-associate. Another limitation of the study is the insect expression system, which results in a reduced N-linked glycan compared with the glycan attached when FXII is secreted from the liver in humans. A limitation of the FXII^FnII^ structural information pertaining to Zn^2+^ ion binding is that the concentrations of Zn^2+^ ions used in the crystallization experiment are higher than would be encountered by FXII in blood plasma.

In conclusion, these two FXII N-terminal domain structures serve to unify the large body of biochemical studies describing the functions of individual domains and provides a scaffold to understand the interactions of FXII with polyanions and Zn^2+^ ions.

## Supplementary Material

PDB reference: FXII^HC5^, 8os5

PDB reference: FXII^FnII^, complex with Zn^2+^, 7prj

Supplementary Figures and full captions to Supplementary Movies. DOI: 10.1107/S2059798325005297/gi5044sup1.pdf

Supplementary Movie S1. Rocking movie showing the cartoon diagram of the FXIIHC dimer structure with two interlocking torc shapes. DOI: 10.1107/S2059798325005297/gi5044sup2.mp4

Supplementary Movie S2. Rocking movie showing the FXIIHC dimer molecular surface and charged residue clusters. DOI: 10.1107/S2059798325005297/gi5044sup3.mp4

Supplementary Movie S3. Movie showing the conformational change of the FXII FnII latch loop. DOI: 10.1107/S2059798325005297/gi5044sup4.mp4

## Figures and Tables

**Table 1:** Crystallographic data-collection and structure-refinement statistics Values in parentheses are for the highest resolution shell.

	FXII^HC5^	FXII^FnII^–Zn^2+^
Data collection		
Space group	*I*2_1_2_1_2_1_	*P*2_1_2_1_2_1_
Temperature (K)	93	100
*a*, *b*, *c* (Å)	144.5, 144.6, 155.9	26.3, 40.9, 45.3
α, β, γ (°)	90, 90, 90	90, 90, 90
Resolution (Å)	77.97–3.4	22.78–1.2
*R*_merge_†	0.179 (1.03)	0.121 (0.395)
〈*I*/σ(*I*)〉	7.1 (2.1)	7.5 (2.2)
Completeness (%)	94.2 (66.2)	98.1 (97.0)
Multiplicity	6.4 (6.4)	4.5 (4.6)
CC_1/2_	0.994 (0.585)	0.993 (0.822)
Total/unique reflections	13995 (701)	15507 (778)
Structure refinement		
*R*_work_‡	0.260 (0.36)	0.174 (0.224)
*R*_free_	0.320 (0.49)	0.192 (0.240)
R.m.s. deviations		
Bond lengths (Å)	0.013	0.005
Bond angles (°)	2.05	0.768
*B* factor (Å^2^)	118.9	9.6
Ramachandran plot		
Favoured (%)	91.6	98.11
Allowed (%)	8.0	1.89
Outliers (%)	0.4	0
PDB code	8os5	7prj

† *R*_merge_ = 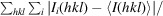

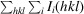
, where *I*(*hkl*) is the observed intensity and 〈*I*_*hkl*_〉 is the average intensity of multiple observations calculated from symmetry-related reflections.

‡ *R*_work_ = 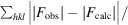

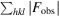
, where *F*_obs_ and *F*_calc_ are the observed and calculated structure factors, respectively. *R*_free_ is computed as for *R*_work_, but for only a randomly selected 5% of the reflections, which were omitted during refinement; it was calculated using *Phenix*.

**Table 2:** SEC-SAXS data collection and processing for FXII^HC5^

Data collection	
Instrument	B21, Diamond Light Source
Beam diameter (µm)	1.0 × 0.25
Wavelength (Å)	0.9408
Detector	EIGER X 4M
*q*-range (Å^−1^)	0.0045–0.34
Exposure time per frame (s)	3
Concentration (mg ml^−1^)	1.5
Temperature (K)	288.15
Guinier analysis	
*qR*_g_ range	0.0045–0.15
*R*_g_ (Å)	28.03
*q*_max_*R*_g_	1.25
*p*(*r*) analysis	
*D*_max_ (Å)	105
*DENSS*	
*R*_g_ (Å)	27.61
Resolution (Å)	32
Volume (Å^3^)	174000
χ^2^	1.48
Molecular weight (kDa)	
*SAXSMoW* calculated	51
