# Supplementary material for: Crystal structure of coagulation factor XII N-terminal domains 1–5
Source: Acta Crystallogr D Struct Biol. 2025 Jun 27;81(Pt 7):380–93. doi: 10.1107/S2059798325005297 (PMC12216678; doi:10.1107/S2059798325005297)
Supplement: Supplementary file 1 [file d-81-00380-sup1.pdf]

STRUCTURAL
BIOLOGY

Volume 81 (2025)

Supporting information for article:

Crystal structure of coagulation Factor XII N-terminal domains 1–5

Muhammad Saleem, Chan Li, Bubacarr G. Kaira, Alexander K. Brown, Monika Pathak, Shabir Najmudin, Nathan Cowieson, Ingrid Dreveny, Clare Wilson, Aleksandr Shamanaev, David Gailani, Stephanie A. Smith, James H. Morrissey, Helen Philippou and Jonas Emsley

A

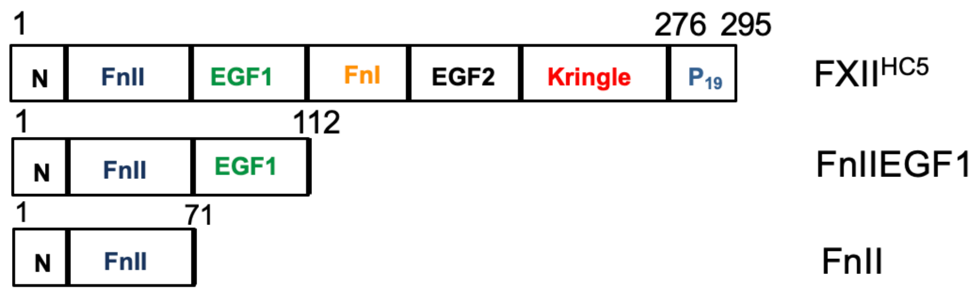

B

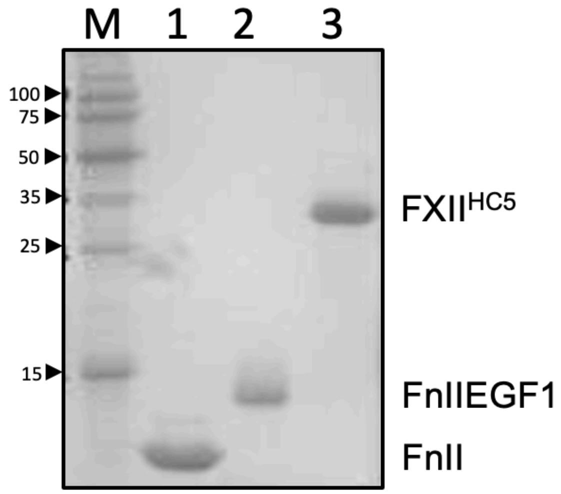

Figure S1. A. Factor XII domain boundaries and constructs used to generate recombinant purified FXII N-terminal domain combinations. B. Coomassie stained SDS PAGE gels of 1. $FXII^{HC5}$, 2. NFE, 3. NF and M denotes the marker with molecular weights annotated in kDa. All fragments were expressed at high levels using the Drosophila expression system (DES) and purified from media.

Protein List

Protein Group	Protein ID	Accession	-10lgP	Coverage (%)	#Peptides	#Unique	PTM	Avg. Mass	Description
1	428	sp|P00748|FA12_HUMAN	338.55	37	61	61	Y	67792	Coagulation factor XII OS=Homo sapiens GN=F12 PE=1 SV=3
total 1 proteins									

sp|P00748|FA12_HUMAN

| Protein Coverage | Supporting Peptides |

Protein Coverage:

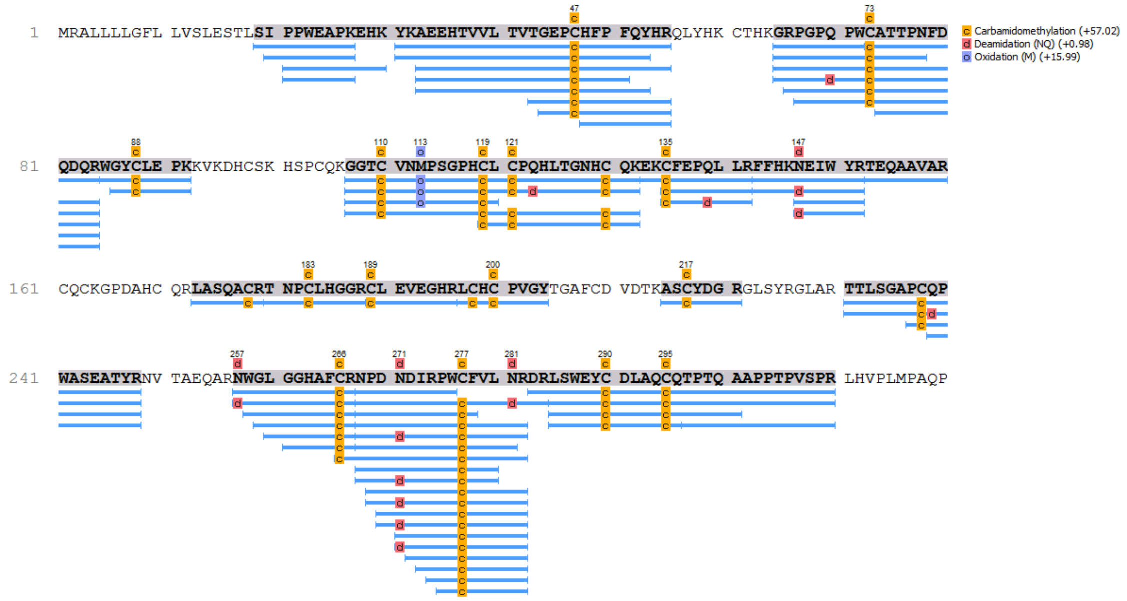

Figure S2. Biochemical characterisation recombinant FXII^{HC} by mass Spectrometry. The sequence of the recombinant FXIIHC was confirmed at the biological mass spectrometry facility, University of Leeds. For this purpose, purified FXII^{HC} sample was run on SDS-PAGE and gel was stained by Expedeon InstantBlue Comassie ready for 30 minutes and de-stained with several changes of MilliQ H2O overnight. The respective band was excised from the gel, treated with trypsin and applied to mass spectrometry. Tryptic digestion produced numerous fragments which were analysed and the resultant peptides were shown from the FXII sequence as shown above.

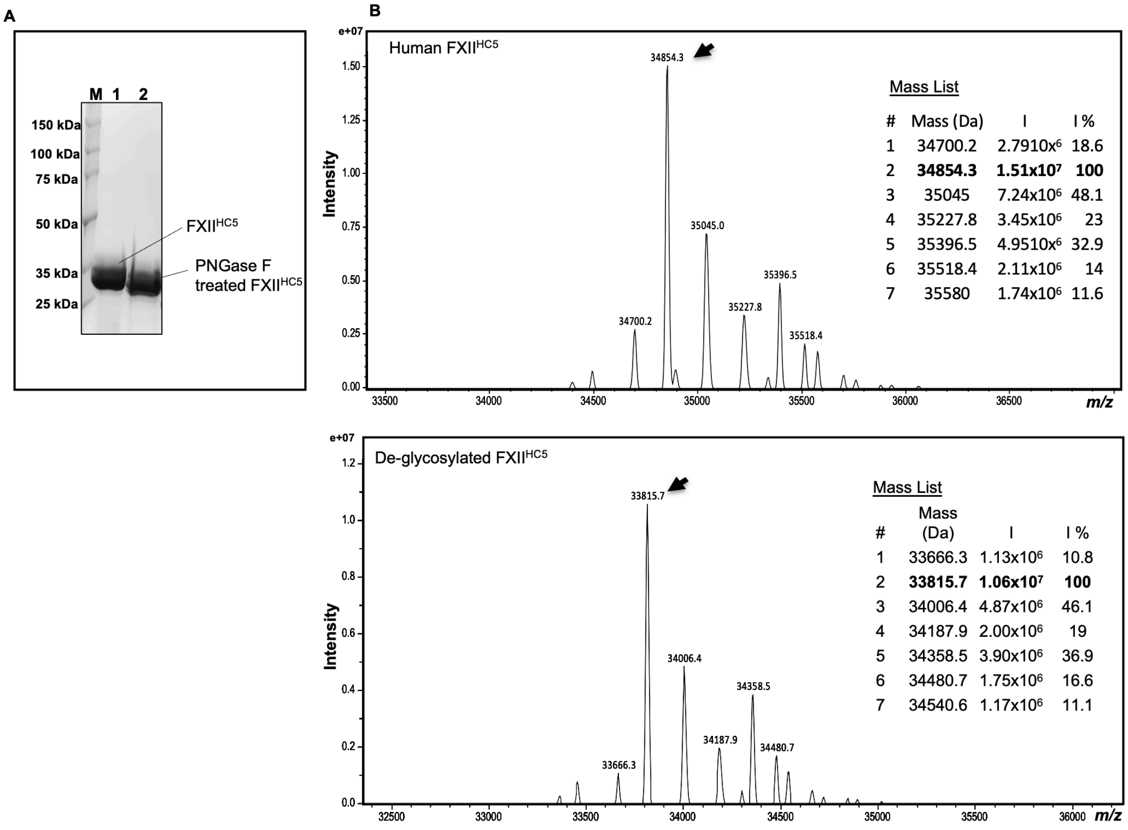

A
M 1 2
150 kDa
100 kDa
75 kDa
50 kDa
35 kDa
25 kDa
FXII^{HC5}
PNGase F treated FXII^{HC5}
B
Human FXII^{HC5}
Intensity
e+07
m/z
34854.3
35045.0
34700.2
35227.8
35396.5
35518.4
Mass List
Mass (Da) I I %
1 34700.2 $2.7910x^{6}$ 18.6
2 34854.3 $1.51x10^{7}$ 100
3 35045 $7.24x10^{6}$ 48.1
4 35227.8 $3.45x10^{6}$ 23
5 35396.5 $4.9510x^{6}$ 32.9
6 35518.4 $2.11x10^{6}$ 14
7 35580 $1.74x10^{6}$ 11.6
De-glycosylated FXII^{HC5}
Intensity
e+07
m/z
33815.7
34006.4
34358.5
34187.9
34480.7
33666.3
Mass List
Mass (Da) I I %
1 33666.3 $1.13x10^{6}$ 10.8
2 33815.7 $1.06x10^{7}$ 100
3 34006.4 $4.87x10^{6}$ 46.1
4 34187.9 $2.00x10^{6}$ 19
5 34358.5 $3.90x10^{6}$ 36.9
6 34480.7 $1.75x10^{6}$ 16.6
7 34540.6 $1.17x10^{6}$ 11.1

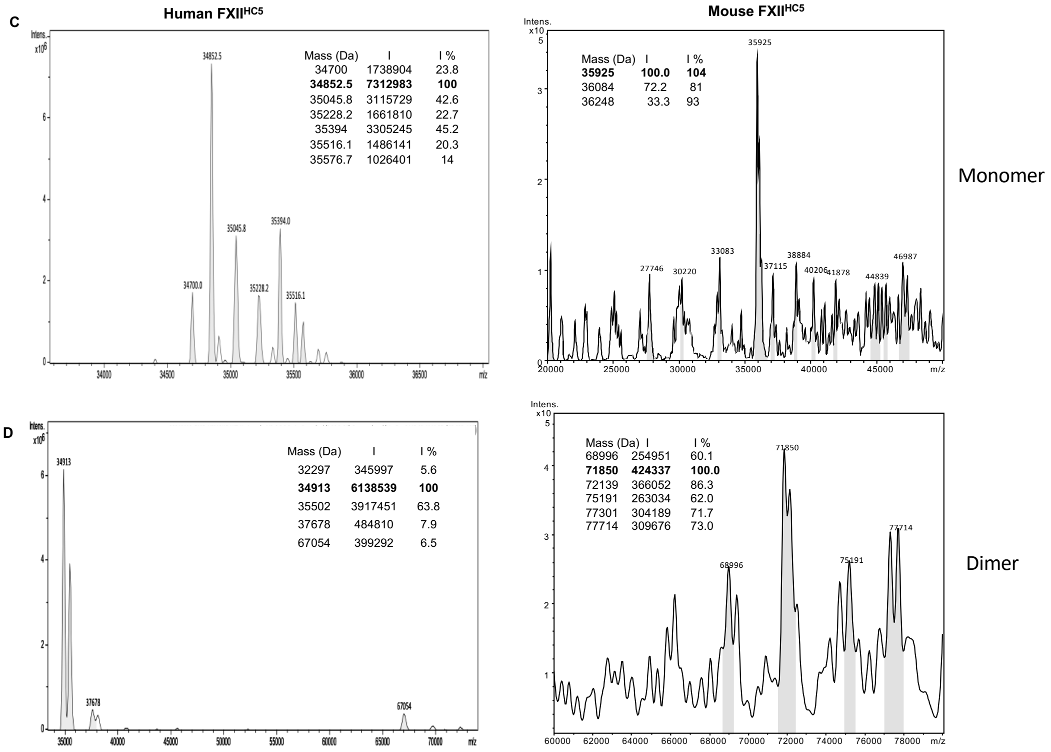
C
Human FXII^{HC5}
Mass (Da) I I %
34700 1738904 23.8
34852.5 7312983 100
35045.8 3115729 42.6
35228.2 1661810 22.7
35394 3305245 45.2
35516.1 1486141 20.3
35576.7 1026401 14
Mouse FXII^{HC5}
Mass (Da) I I %
35925 100.0 104
36084 72.2 81
36248 33.3 93
Monomer
D
Mass (Da) I I %
32297 345997 5.6
34913 6138539 100
35502 3917451 63.8
37678 484810 7.9
67054 399292 6.5
Mass (Da) I I %
68996 254951 60.1
71850 424337 100.0
72139 366052 86.3
75191 263034 62.0
77301 304189 71.7
77714 309676 73.0
Dimer
m/z

Figure S3. Glycan content of recombinant $FXII^{HC5}$. A. Purified human $FXII^{HC}$ sample was run on SDS-PAGE and gel was stained by Brilliant Coomassie G and de-stained with H2O. The respective band was excised from the gel, treated with trypsin and applied to mass spectrometry. Resulting peptides were analysed by MALDI-MS and MS/MS using a Bruker ultrafleXtreme TOF/TOF. MS/MS spectra were acquired and matched to the UniProt protein database as well as provided amino acid sequence of $FXII^{HC5}$ using Mascot software to match proteins. PNGaseF (NEB) was used for digestion of native recombinant human $FXII^{HC5}$ protein according to protocol for non-denaturing reaction conditions provided by supplier. Overall, the reaction was set up in absence of any detergent or denaturing agents, using a 10-fold higher enzyme/protein ratio than recommended for denaturing conditions. The reaction was incubated for 6 hours at 37^0 C and afterwards the samples were analysed on SDS PAGE. B. These studies were performed at university of York Mass Spectrometry (MS) facility. For denaturing MS, protein was diluted 1:20 into aqueous 50% acetonitrile containing 1% formic acid. For native mode mass spectrometry, protein was buffer exchanged using Amicon MW spin filters into 1M ammonium acetate pH 7.0. Protein solution was infused at 3 ml/min into a Bruker maXis qTOF mass spectrometer via an electrospray ionisation source. Source conditions, and ion optic parameters were adjusted to favour detection of native protein states. Specifically: Dry gas, 250°C at 6 l/s; Funnel RF, 400 Vpp; Multipole RF, 200 Vpp; Quadrupole low cutoff 1200 *m/z*; Quadrupole ion energy offset, 3 eV; Prepulse storage, 50 ms; Transfer time, 160 ms. Spectra were summed over 1 min acquisitions at 0.1 Hz. Data were smoothed (0.2 Da, 1 cycle, Gauss) maximum entropy deconvolution to average masses at 200 resolution. Separate deconvolutions were performed for the charge state regions resulting from the protein monomer and dimer and in total cover the mass range 20-80 kDa. Data acquisition was performed using Bruker Hystar and oTof control (version 4.1). Peak picking and spectral processing were undertaken using Bruker DataAnalysis software (version 4.4). C, D. MS analysis of a human and mouse FXIIHC5 protein under native conditions. The MS parameters were optimised specifically for the *m*/*z* window of different forms of protein and the mass spectra was deconvoluted. The protein spectra along-with mass lists is shown where both monomer and dimer protein forms are separated in *m*/*z* space in the 20000–80000 range.

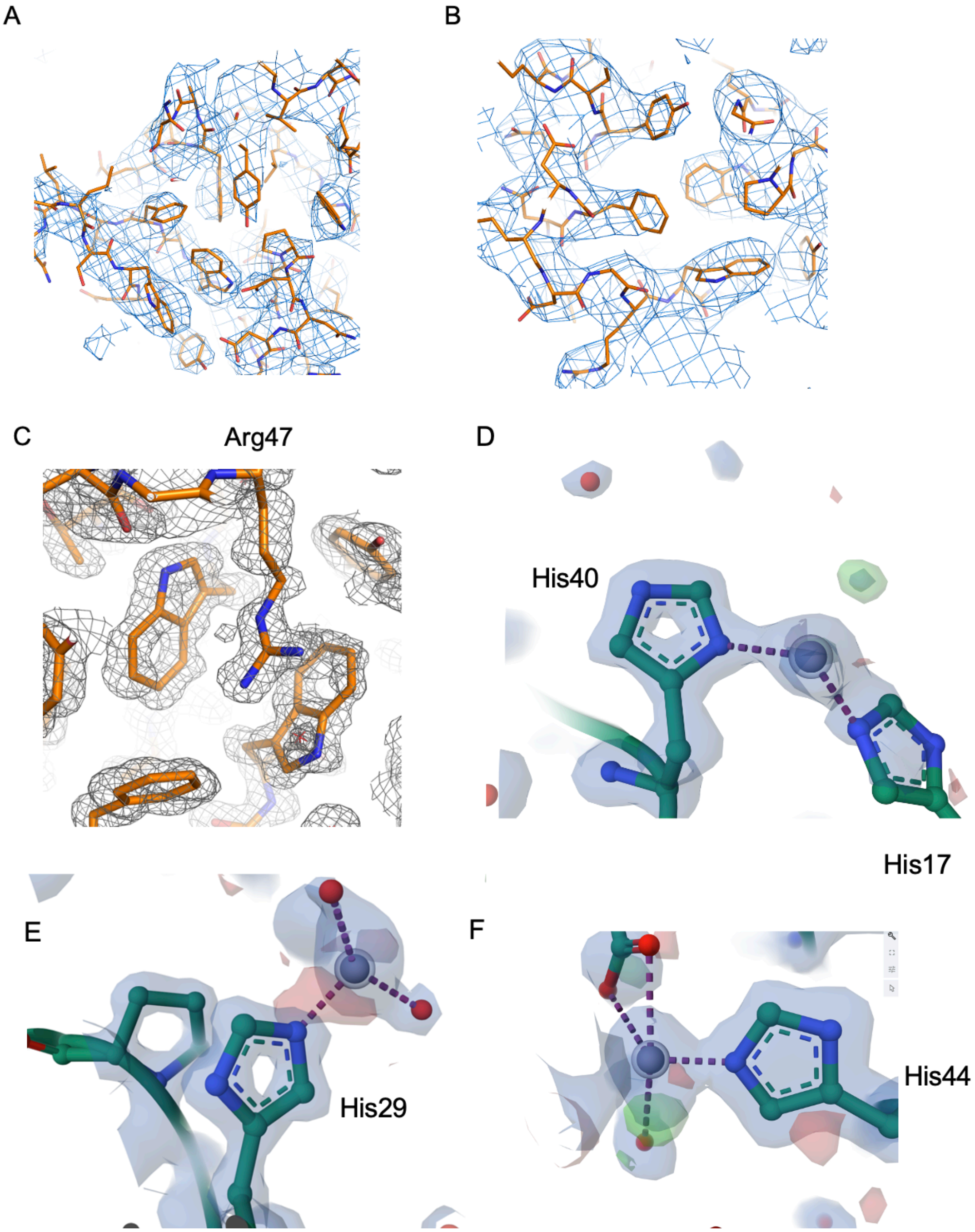

Figure S4. Electron density map for the FXII^{HC} and FnII structures. 3.4Å resolution 2F_{obs}-F_{calc} map contoured at 1σ for the **(A)**, FXII^{HC} kringle and **(B)**, FXII^{HC} FnII domain. The map was calculated using phenix and rendered in Pymol. **(C,D,E,F)** 1.2Å resolution 2F_{obs}-F_{calc} map contoured at 1.3σ for the isolated FXII FnII domain calculated using phenix and rendered in Pymol.

Movie S1.

Rocking movie showing the cartoon diagram of the $FXII^{HC}$ dimer structure with two interlocking torc shapes. Colors are FnII domain (blue), linker (cyan), EGF1 (green), FnI (orange), EGF2 (black), kringle (red). The FnII and the kringle domain forming a head to tail intramolecular interaction and key residues are shown as sticks. This view shows a triangular shape with close relative positioning of the kringle domains at the top. The lysine binding site residues are shown as sticks and EGF1 residue K81 is shown as spheres (green).

Movie S2.

Rocking movie showing the $FXII^{HC}$ dimer molecular surface and charged residue clusters. The charged molecular surface is transparent (blue=positive, red=negative). Beneath the surface is a $FXII^{HC}$ cartoon diagram showing the dimer with the two L-shaped FnII-EGF1-FnI polypeptides colored gold or grey. Clusters of surface exposed residues are shown as sticks and spheres colored blue=arginine or lysine, cyan=histidine, light blue=asparagine or glutamine.

Movie S3.

Movie showing the conformational change of the FXII FnII latch loop. Cartoon diagram of the isolated FnII domain colored by secondary structure, β-sheets (pink), α-helix (cyan). A morph was calculated using pymol animating the conformational change between the isolated FnII and $FXII^{HC}$ FnII structures. Latch loop residue R47 (light blue) interacts with the FnII cation binding site shown as sticks (pink). Their animation shows the switch in occupancy of the FnII cation binding site to P48 (green) in the $FXII^{HC}$ structure where R47 is involved in interactions with the kringle domain. Electrostatic interactions are shown as purple dotted lines.
